# Narrative identity in addictive disorders: a conceptual review

**DOI:** 10.3389/fpsyg.2024.1409217

**Published:** 2024-06-17

**Authors:** Valentina Deriu, Daniela Altavilla, Ines Adornetti, Alessandra Chiera, Francesco Ferretti

**Affiliations:** Cosmic Lab, Department of Philosophy, Communication and Performing Arts, Roma Tre University, Rome, Italy

**Keywords:** addictive disorders, narrative identity, self, autobiographical narrative, narrative analysis, narrative coherence, agency, emotions

## Abstract

Narrative identity allows individuals to integrate their personal experiences into a coherent and meaningful life story. Addictive disorders appear to be associated with a disturbed sense of self, reflected in problematic and disorganized self-narratives. In recent literature, a growing body of research has highlighted how narrative approaches can make a dual contribution to the understanding of addiction: on the one hand, by revealing crucial aspects of self structure, and, on the other, by supporting the idea that addiction is a disorder related to unintegrated self-states in which dissociative phenomena and the resulting sense of ‘loss of self’ are maladaptive strategies for coping with distress. This conceptual review identified the main measures of narrative identity, i.e., narrative coherence and complexity, agency, and emotions, and critically examines 9 quantitative and qualitative studies (out of 18 identified in literature), that have investigated the narrative dimension in people with an addictive disorder in order to provide a synthesis of the relationship between self, narrative and addiction. These studies revealed a difficulty in the organization of narrative identity of people with an addictive disorder, which is reflected in less coherent and less complex autobiographical narratives, in a prevalence of passivity and negative emotions, and in a widespread presence of themes related to a lack of self-efficacy. This review points out important conceptual, methodological and clinical implications encouraging further investigation of narrative dimension in addiction.

## 1 Introduction

Pathological addictions encompass a range of disorders that are characterized by abnormal and persistent substance use or the recurrent engagement in a specific behavior that causes significant distress to the person by affecting multiple domains of the individual’s personal and social experience (American Psychiatric Association, 2013). The latest edition of the Diagnostic and Statistical Manual of Mental Disorders (DSM-5; American Psychiatric Association, 2013) has highlighted some overarching criteria underlying addictions that are common to substance use disorders (SUDs), which include substance abuse and dependence (either alcohol or drugs), and non-substance addiction-related disorders, which include Gambling Disorder (GD) (Caretti and La Barbera, 2004; Gabbard, 2015; Rash et al., 2016). These criteria include loss of voluntary control and maladaptive impulsive behavior over the object of addiction: people with an addictive disorder continue to use or engage in the behavior despite negative consequences for their family and interpersonal relationships, and despite potential problems in their work environment (Wareham and Potenza, 2010; Rash et al., 2016). These elements seem to indicate difficulties in the underlying mechanisms of decision-making processes, concerning how people with an addictive disorder evaluate the risks and benefits of a choice. In this vein, several investigations have shown that people with SUDs or GD cannot control their irresistible urge for an immediate reward, e.g., the satisfaction of using drugs or immediate gain, and do not consider possible negative consequences in the future, i.e., temporal discounting (Ainslie, 2001; Bechara et al., 2002; Bechara and Damasio, 2002; Reynolds, 2006; Brewer and Potenza, 2008; Mestre-Bach et al., 2016; Jimenez-Murcia et al., 2017; Steward et al., 2017). Brain imaging studies investigating the decision-making process in GD and SUDs have shown reduced activity in the ventromedial prefrontal cortex and ventral striatum, which is associated with impairment in cognitive inhibitory control and reward-related decision-making processes in both groups compared to controls (e.g., Potenza et al., 2003; Reuter et al., 2005; Tanabe et al., 2007; Potenza, 2009). Another factor that plays an important role in the consideration of addictive disorders is the presence of craving, defined as a strong motivation or irresistible desire to use (Sayette et al., 2000; Hasin et al., 2013; Sayette, 2016; Antons et al., 2020). In addition to being one of the main diagnostic criteria for SUDs, considerable evidence has shown that craving is also relevant for non-substance addictions, such as GD, and appears to be associated with more severe disease (for a review, see Mallorquí-Bagué et al., 2023).

The study of addiction spans many disciplines and perspectives, including clinical psychology, cognitive neuroscience, and sociology, which have contributed to identify specific behavioral, cognitive, and neurobiological patterns of symptomatology. Notwithstanding, there is a “puzzling” lack of agreement about the nature of the phenomenon (Pickard, 2018, p. 741; Pickard, 2020). Traditionally, theoretical models that have attempted to explain addiction can be grouped into the following main frameworks: on the one hand, the biomedical or brain disease model (e.g., Leshner, 1997; Volkow and Morales, 2015; Koob and Volkow, 2016), according to which addiction is a biological dysfunction linked to genetic factors over which the individual has no control (Adinoff, 2004; Le Moal and Koob, 2007). However, this model seems to ignore some important psychological and social aspects that are crucial for understanding the phenomenon. On the other hand, the moral model assigns responsibility to individuals who have chosen the rewards of drug use or gambling (Wilbanks, 1989). Because this perspective associates addiction with a negative moral connotation, i.e., a kind of “impurity” of the individuals involved, it has recently been challenged by other, more neutral perspectives, the so-called “choice model,” which applies the principles of ordinary choice behavior to addiction without stigmatizing it (Heyman, 2009; Foddy and Savulescu, 2010; for a discussion see Kennett et al., 2013; Pickard, 2017; Rise and Halkjelsvik, 2019). Finally, cognitive-behavioral and psychodynamic approaches highlight the role of learning and interpersonal relationships in addictive disorders. In particular, Lewis (2015, 2017) explains the addiction with the “learning model,” according to which addiction is associated with a “repeated experience” that develops into a habit through the establishment of learned patterns of reward-seeking behavior (Lewis, 2017, p. 10). Psychodynamic approaches and attachment theory, instead, underline the importance of early relational experiences. Specifically, people with dysfunctional and traumatic relational experiences that have not allowed them to build a solid self with a stable self-esteem, a reliable representation of the other, low mentalization abilities, and an effective emotional regulation might act the addictive behavior as a maladaptive strategy against suffering in order to control affects as shame, depression, anger and guilt (e.g., Kohut, 1971; Fonagy and Target, 1997; Bromberg, 1998, 2006; Johnson, 1999; Flores, 2004; Rothschild and Gellman, 2009; Schindler, 2019; Liese et al., 2020). Consistently, Khantzian (1985) suggested the “self-medication hypothesis” to describe addictive disorders.

Within this background, several investigations showed that the experience of craving in both GD and SUDs is associated with deficient emotion regulation strategies, dissociation, and impairments in self-control as well as in decision-making processes such as pursuit persistence and delay discounting, suggesting a more severe condition and a greater likelihood of relapse (De Castro et al., 2007; Tárrega et al., 2015; Serre et al., 2018; Ciccarelli et al., 2019; Schluter and Hodgins, 2021; Velotti et al., 2021). These features seem to suggest that addictive disorders are characterized by an unintegrated self, particularly affecting the systems that mediate the capacity for self-determination, self-governance, and self-concepts related to one’s social role (e.g., Reith and Dobbie, 2012; McConnell, 2016; Pickard, 2020). In this regard, the analysis of patients’ self-structure could be particularly important for understanding the different factors that might contribute to explaining the phenomenon of addiction. In this paper we will focus on this aspect. The self is a multifaceted concept that refers to a set of physical, psychological, and cultural characteristics that provide individuals with a sense of unity, purpose, and meaning throughout the lifespan (e.g., Erikson and Erikson, 1997; Habermas and Bluck, 2000; Pasupathi et al., 2009; McAdams and McLean, 2013; Marraffa and Meini, 2016; Marraffa and Meini, 2021). Recent research has observed that the experience of addiction is associated with a loss of connection to reality and a change in one’s identity, accompanied by dissociative phenomena in which people with an addictive disorder feel that an alien self is controlling their actions (Keane, 2002; McCormick et al., 2012; Reith and Dobbie, 2012; Dill and Holton, 2014; Schluter and Hodgins, 2019). Specifically, dissociative experiences in addiction refer to both a disrupted integration of memories, emotions, experiences, and thoughts, and a painful self-awareness related to the stigmatization of the social identity (e.g., Corrigan et al., 2009; Reith and Dobbie, 2012; Schüll, 2012; Matthews et al., 2017; Pickard, 2020; Rogier et al., 2021b). Furthermore, it has been suggested that addictive disorders are characterized by conflictual phenomena between a predominant strong desire to use/behave and the self-control system, resulting in a loss of agency and self-efficacy (MacKillop et al., 2011; Dill and Holton, 2014).

In cognitive psychology and personality research (e.g., Riessman, 1990; Giddens, 1991; McLean et al., 2007; McAdams and McLean, 2013), it has been hypothesized that a fruitful way of framing the structure of the self is through the narrative dimension. In this perspective, the self, particularly its diachronic dimension, is interpreted as an evolving life story through which individuals give meaning to their existence by organizing experiences into a coherent and unified representation in order to develop a stable sense of self, i.e., narrative identity (Dennett, 1993; Gallagher, 2000; Singer, 2004; McAdams and McLean, 2013). Borrowing the Jamesian tradition, narrative identity is configured as a selfing process resulting in an autobiographical narrative, constructed by the narrator/Me through the integration of complex plots involving past personal episodes, key experiences (low, high, or turning points), relationships with other characters, self-referential future episodes, and psychological states (McAdams, 1989, 1996, 2001; Conway and Pleydell-Pearce, 2000; McAdams and Olson, 2010; Marraffa and Meini, 2016; Marraffa and Meini, 2021), and which requires autobiographical remembering. Therefore, according to this model, autobiographical memory serves the core function of providing the building blocks for the construction of the self, as it enables individuals to recall information about the what, when, and where of self-referential past episodes, as well as to plan future behaviors, thus contributing to the configuration of one’s life story as told from a subjective point of view (Tulving, 1985; Habermas and Bluck, 2000; Singer et al., 2013).

Relevant to the aim of this paper, according to several authors, the way in which individuals recall and narrate their personal experiences and integrate them into an overall life story seems to reveal important cognitive and affective aspects of the cohesion of self (McAdams and McLean, 2013). In particular, recent research has shown that specific features of self-narratives can be associated with certain psychopathological conditions (e.g., Vanden Poel and Hermans, 2019). It is worth emphasizing this point, because it follows that research on narrative identity can make a significant contribution not only to the theoretical framing of the sense of self, that is associated with greater psychological well-being (e.g., McLean et al., 2010; Reese et al., 2017), but also to the understanding of psychological disorders and, consequently, to the development of effective interventions (Singer et al., 2013). As addiction appears to be characterized by a self prone to fragmentation (e.g., Reith and Dobbie, 2012), the study of personal narratives of people with an addictive disorder could be an important contribution to understanding the fragile structure of the self in addiction.

Against this background, the aim of this paper is to propose a conceptual analysis of the existing literature on the narrative dimension in addictions, in order to highlight the role of narrative as a tool for investigating the self in addictive disorders. The article is divided into the following sections: in the “Measures of narrative identity” section, we provide a conceptual analysis review of the key variables in autobiographical narratives that have been associated with disturbances in the self dimension (McLean et al., 2020); in the “Studies on narrative identity in addictions” section, we review the existing literature that has examined these variables in addictive disorders through narrative-based approaches. In the final section, “Discussion,” we offer an examination of these studies taking into account the main features that characterize addiction, and we will argue for further research on narrative identity in addiction in order to better understand the dynamics of this disorder.

## 2 Measures of narrative identity

### 2.1 Structural variables: narrative coherence and narrative complexity

The structural elements of a narrative refer to the overall architecture of a story, including the organization of the temporal order and spatial location of events, and the causal connection between them, i.e., *narrative coherence*, and the number of relevant details in the story, i.e., *narrative complexity* (e.g., McLean et al., 2020). Indeed, the individual’s ability to coherently connect personal experiences across space and time in an overall representation, an ability that emerges particularly during adolescence (e.g., Habermas and Bluck, 2000), seems to be associated to a greater and more stable structure of the self (e.g., Linde, 1993; Habermas and Bluck, 2000; Habermas and de Silveira, 2008; Herman, 2013; Köber and Habermas, 2017; Köber et al., 2019). Thus, narrative coherence can represent a crucial measure for the assessment of narrative identity (e.g., Adler et al., 2018).

More specifically, narrative coherence is a structural property that reflects the ability to organize a sequence of events, establishing causal and temporal links between them in the construction of a life story (Richardson, 2000; Reese et al., 2011; King et al., 2014), with the connected events corresponding to personal episodes, so that the resulting personal narrative provides both a naive listener and the narrator with an understanding of the meaning of the events (Reese et al., 2011). According to one prominent view, narrative coherence is a multidimensional construct that is the product of at least three interrelated aspects that correspond to the operational criteria of analysis: the temporal dimension, which refers to the narrator’s ability to determine when and in what order (chronology) personal events took place; the causal-motivational coherence, i.e., the connection between the events that make up the sequence (the plot of the story) and the motivations behind them; the thematic-evaluative dimension, which corresponds to the identification of how certain themes can be applied to these events and influence the self (Habermas and Bluck, 2000; Reese et al., 2011). Precisely because narrative coherence manages to capture the entire organization of the life story, revealing individuals’ ability to organize and make sense of their personal experiences, some authors have suggested that the degree of narrative coherence is an important predictor of psychological well-being (e.g., for a review see Baerger and McAdams, 1999; Tuval-Mashiach et al., 2004; Lysaker and Lysaker, 2006; McAdams, 2006; Waters and Fivush, 2015; Adler et al., 2016; Vanden Poel and Hermans, 2019). In this direction, although there are huge differences in the extent to which healthy individuals construct coherent life stories, also considering that they may be mediated by several factors (including social and relational ones, e.g., Iftode et al., 2022), it seems that such gradual differences, in terms of more or less coherent autobiographical narratives, may be related to several psychological aspects: more coherent life stories have been related to life satisfaction and lower levels of depressive traits (e.g., Baerger and McAdams, 1999), whereas less coherent autobiographical narrative has been associated with impairment in self integration, i.e., a fragmented sense of self with parts of oneself perceived as alien (e.g., Vanderveren et al., 2021), and with psychopathological traits (e.g., Semerari, 2001; Lysaker et al., 2002; Lysaker and Lysaker, 2006; Adler, 2012; Vanderveren et al., 2019; Mitchell et al., 2020). For example, in a longitudinal study of adolescent narratives, Mitchell et al. (2020) have found that coherence, particularly the causal subcomponent in the narration of turning points, was positively associated with greater life satisfaction, and negatively associated with rumination and depression symptoms, suggesting that more coherent narratives of life experiences may play a critical role in achieving greater psychological well-being. In support of this view, other investigations have shown that lower levels of thematic coherence in personal narratives are also associated with depressive symptoms and anxiety (Vanden Poel and Hermans, 2019; Vanaken et al., 2021). Of relevance to our purpose is the suggestion that where there are difficulties in the elaboration of narrative coherence that affect at least one of the three dimensions (temporal, causal, and thematic) and result in a disrupted autobiographical story, there also appears to be a disruption in the cohesion of the self. Within the clinical literature, this hypothesis has been supported by some studies of the narrative dimension in different psychopathologies, such as schizophrenia (e.g., Lysaker et al., 2002; Raffard et al., 2010; Saavedra, 2010; Allé et al., 2015; Holm et al., 2016; Goldberg, 2023; for a discussion see Lysaker and Lysaker, 2006; Adornetti and Ferretti, 2021; Benítez-Burraco et al., 2023), autism (e.g., Diehl et al., 2006; Goldman, 2008; King et al., 2014; Samra, 2016; Ferretti et al., 2018; Wantzen et al., 2021; Adornetti et al., 2023; Harvey et al., 2023), and personality disorders (e.g., Westen and Cohen, 1993; Bradley and Westen, 2005; Adler et al., 2012; Lind et al., 2019; for a review, see Lind et al., 2020). For example, the findings of Allé et al. (2015) revealed that the autobiographical narratives of patients with schizophrenia were less coherent than those of controls in the temporal, causal-motivational, and thematic subcomponents, supporting the link between lower coherence and a disordered structure of the self. With regard to personality disorders, Adler et al. (2012) analyzed the global coherence of personal narratives of patients diagnosed with borderline personality disorder (BPD) and a control group, showing that the former were significantly different from those of individuals without the disorder, and that coherence was significantly negatively correlated with several measures of BPD. The authors concluded that impaired cohesion of the self in borderline disorder could also be interpreted in terms of difficulties in constructing a coherent life story (*ibid.*). Because these psychopathologies appear to share some similar features of addiction, such as fragility in the structure of the self and dissociative phenomena (Keane, 2002; Reith and Dobbie, 2012), the aforementioned findings highlight the importance of examining narrative coherence in addictive disorders as well.

As mentioned above, another crucial structural element that can reflect important information on narrative identity construction is narrative complexity. In general, individuals engage in reflective processes to construct their life story, connecting thoughts, emotions, and personal events and reasoning about how they have influenced changes in the self: the degree of complexity of these cognitive and affective processes can result in simple or more sophisticated autobiographical narratives (McAdams, 1993; McAdams et al., 2004; Lysaker et al., 2017; Lind et al., 2019). At the narrative level, complexity coincides with the way in which all the core elements of a story, i.e., subjects, objects, and events located in space and time, are provided with relevant details in terms of the identification of characters’ emotions, thoughts, and motivations, and the degree of intricacy of the story’s plot, i.e., the elaboration of how events are temporally and causally related (e.g., McAdams et al., 2004; Rowlands et al., 2021). Therefore, some scholars have suggested that different levels of narrative complexity may reflect different levels of integration of narrative identity, shedding light on individuals’ abilities to reason about causal connections between personal experiences, i.e., elaborating explanations and motivations behind events and why they are linked, and to introspectively reflect on emotions and thoughts (McLean and Pratt, 2006; Lind et al., 2019). Like coherence, complexity has also been analyzed in the narratives of people with different psychopathologies characterized by a disorder of the structure of the self. In this regard, although several studies have shown, compared to control groups, a lower narrative complexity in some psychopathologies, e.g., schizophrenia (Saavedra, 2010), in other clinical populations, e.g., in borderline personality disorder (BPD), empirical studies have not found difficulties in the elaboration of complex self-narratives, but only in the narratives of the others, i.e., where the patients were asked to describe other people’s life stories (e.g., parents) (Lind et al., 2019, 2020). However, as shown by Lind et al. (2019), at the lowest level of the continuum, the elaboration of motivations about a particular event can be very vague. Interestingly, they also provided an example of a participant who, when asked to explain how leaving home affected her life, responded, “Well, maybe something about the economy, but I’m not really sure.” (*ibid.*, p. 232). On the contrary, higher levels of narrative complexity seem to be associated with more developed reflective skills and a more structured self (e.g., McLean and Pratt, 2006).

In summary, these studies suggest that unintegrated self-states, which are at the core of several of the psychopathologies mentioned (e.g., schizophrenia), are reflected in less coherent and impoverished self-narratives. For this reason, the analysis of narrative structure, especially global coherence, could contribute to a fruitful study of the self, even in addictive disorders.

### 2.2 Motivational theme: agency

Over the past few decades, another key narrative marker that has attracted considerable interest in the psychological literature for assessing self-related aspects is agency (e.g., Woike and Polo, 2001; Adler et al., 2012). Agency plays a crucial role in shaping narrative identity by providing individuals with a sense of purpose and meaning; it serves as a motivational force to achieve personal goals and to perceive oneself as in control of the course of personal events, i.e., self-efficacy (McAdams et al., 1996; Bandura, 2000, 2006; McAdams, 2001; Adler et al., 2008; Lysaker et al., 2010; Adler, 2012; Adler et al., 2012; Lind et al., 2019). Self-efficacy is a facet of self, and specifically of the self-awareness that, we saw in the introduction, appears to be impaired in addictive disorders (e.g., Dill and Holton, 2014). Precisely because agency allows us to capture the motivations and purposes of individuals acting in their life stories, it pertains to the thematic dimension of autobiographical narratives and is often associated with the theme of communion, i.e., the individuals’ motivations for relationship (e.g., attachment, intimacy, love) (McAdams et al., 1996; McLean, 2017; Park and Moon, 2022), even though the role of this latter aspect seems rather controversial (e.g., Gehrt et al., 2023). Thus, in most research, agency is assessed using a thematic coding scheme, e.g., the most common is a 3- or 5-point scale that refers to the whole personal narrative and assesses the extent to which individuals perceive their ability to act and influence the events in their lives (McAdams et al., 1996; Adler et al., 2008, 2012; Adler, 2012). Recently, some scholars have suggested that agency is also reflected in specific linguistic markers, such as first-person singular pronouns, which may represent an index of self-determination and self-efficacy (Van Staden and Fulford, 2004; Nikzad et al., 2023).

It is worth noting that higher levels of agency have been positively associated with mental health and a more stable cohesion of the self, whereas low levels of agency have been observed in several psychopathological conditions, such as depression (Helgeson, 1994; Slaby, 2012). In this regard, several investigations have shown that people with a disrupted narrative identity tend to narrate themselves as less agentic, that is, they perceive themselves passively influencing their lives and unable to control their actions (e.g., Adler et al., 2012). In this sense, a low degree of agency has been observed in the autobiographical narratives of people with a psychopathological condition. For instance, a lack of agency (and causal coherence) was found in turning point narratives of individuals at high psychometric risk for schizophrenia (Hazan et al., 2019). Another recent study by Jensen et al. (2021) has investigated the sense of agency in autobiographical narratives about the past and future of a group of patients with schizophrenia, a group of patients with depressive disorder, and a control group. The results have shown in both clinical groups a reduced agency in the past condition compared to the control group. As with personality disorders, “the experience of self as agentic is often disrupted in borderline personality by a pattern in which impulses are acted upon so immediately that the self is not experienced as the author of the act” and this is reflected in less coherent and interrupted self-narratives (Bradley and Westen, 2005, p. 937). In this vein, Adler et al. (2012) found that the narratives of people with a personality disorder significantly differed from those of control groups in terms of agency other than narrative coherence, reinforcing the view of a relationship between difficulties in constructing a stable sense of self and low agency. In support of this finding, Lind et al. (2019) also have found that patients with personality disorder produced personal narratives with few references to the theme of agency compared to a control group. In general, these studies provide an empirically grounded view of how the analysis of agency in personal narratives can capture important aspects of narrative identity construction, particularly highlighting the functioning of self-efficacy and performance processes that appear to be compromised in both GD and SUDs.

### 2.3 Emotional dimension of narrative identity

The emotional dimension is another fundamental component of narrative identity organization (Horrocks and Callahan, 2006; Habermas, 2015). First and foremost, emotions have a strong influence on the way individuals internalize and recall personal experiences, especially when it comes to significant autobiographical memories: on the one hand, the emotional state of the protagonist of an experience influences the way such an experience is encoded and stored; on the other hand, the emotional content of the event affects the way the experience is retrieved, i.e., the individual’s representation of that experience (e.g., Pascuzzi and Smorti, 2017). Second, and central to the purpose of our argument, emotions influence how individuals reorganize personal experiences to form autobiographical narratives. Therefore, since these autobiographical narratives play a role in evaluating personal experiences, the ways in which these narratives are used to attribute meaning to experiences appear to be to some extent intrinsically emotional (Habermas, 2015). As a result, in addition to the close relationship between emotions and autobiographical memory mechanisms, the way in which individuals understand, reflect on, and manage their emotions, i.e., emotion regulation processes, also turns out to have a significant impact on the organization and narration of a coherent and meaningful life story (Horrocks and Callahan, 2006; Fox et al., 2008; Holland and Kensinger, 2010; Pascuzzi and Smorti, 2017; Velotti et al., 2021). Specifically, due to their subjective nature, self-narratives are simultaneously imbued with the individual’s emotional experiences and the product of a process of meaning attribution to achieve a coherent life story (Horrocks and Callahan, 2006). In light of these considerations, the evaluation of emotions in personal narratives seems to offer a valid strategy for investigating narrative identity.

Some scholars have proposed a quantitative approach to assessing the emotional content of personal narratives, based on the idea that the words used to describe personal experiences can convey important aspects of an individual’s psychological world (e.g., Pennebaker and Seagal, 1999; Pennebaker and Graybeal, 2001; Pennebaker et al., 2003, 2015; Rude et al., 2004; Junghaenel et al., 2008; Minor et al., 2015; Sonnenschein et al., 2018; Mariani et al., 2020). In this sense, Pennebaker and Seagal (1999) have argued that a small number of negative emotion words is associated with repressive coping strategies, with a deficient ability to label and recognize emotions, and with a greater risk of psychological discomfort, whereas an overuse of them seems to indicate a high neurotic risk. As for positive emotion words, a greater use of them together with some negative emotion words in personal narratives, for example in the narration of traumatic events, suggests that individuals acknowledge their problems, regulate their emotions better, and present a greater sense of well-being in general (Pennebaker and Seagal, 1999; Pennebaker and Graybeal, 2001). These claims appear to be supported by other empirical evidence. For example, Sonnenschein et al. (2018) analyzed negative emotion-related words in the narratives of patients with mood and/or anxiety disorders during psychotherapy and found a higher proportion of negative emotion words (e.g., sadness) in group of participants with a depressive disorder compared to the group of participants with an anxiety disorder. Results from Rude et al. (2004) showed that emotion-related words with negative valence were used more often in written personal narratives by students with depression than by control groups. In addition, linguistic analysis of narratives from a group of patients with schizophrenia and two subgroups of people with a schizoaffective disorder revealed that higher proportions of negative emotion-related words and the subcategory of anger-related words were associated with total symptoms in all groups, particularly a high number of anger words was associated with symptoms of distortion (Minor et al., 2015). Moreover, several studies have confirmed that the narratives of people with high levels of alexithymia, i.e., with difficulties in identifying and describing subjective feelings, limited imaginative capacity, and an externally oriented cognitive style that shows a semantic deficit in terms of emotion words (Wotschack and Klann-Delius, 2013), are also at higher risk for physical and mental illness (e.g., Taylor and Bagby, 2004, 2012). In general, given the maladaptive emotion regulation strategies in addictive disorders (e.g., Dill and Holton, 2014), these investigations allow us to consider the analysis of the emotional content of autobiographical narratives, in terms of the number and type of emotion-related words, as an important avenue for studying the affective processes involved in the organization of narrative identity also in addiction.

## 3 Studies on narrative identity in addictions

Although, as we have shown above, the analysis of autobiographical narratives has provided crucial insights for the study of the self in several clinical populations (e.g., schizophrenia, personality disorders, etc.), there are still few studies investigating narrative identity-related variables in addictive disorders. To the best of our knowledge, out of a total of 18 studies identified in literature that explored narratives of addiction, only two studies have explicitly examined structural features in personal narratives of individuals diagnosed with an addictive disorder: one study focused on the assessment of narrative global coherence (Canali et al., 2021), the other on narrative complexity and its relation to recovery (Rowlands et al., 2021). As for the second variable we considered, namely agency in the framework of narrative identity, only a few studies have examined it in self-narratives of people with an addictive disorder (Altavilla et al., 2020; Canali et al., 2021) and in recovery context (Rowlands et al., 2019; see also an exploratory stud by Rowlands et al., 2020), while other investigations have proposed to analyze agency in autobiographical narratives in addiction using a sociological approach based on actor-network theory (Törrönen and Tigerstedt, 2018; Törrönen et al., 2020; Törrönen, 2023). Finally, one study has analyzed the emotional dimension in autobiographical narratives of people with GD (Altavilla et al., 2020). These investigations adopted a top-down approach by evaluating the selected narrative variables, i.e., coherence, complexity, agency, and emotion, in autobiographical narratives using a qualitative (Rowlands et al., 2019, 2021; Canali et al., 2021) or a quantitative (Altavilla et al., 2020) analysis. In this section, we briefly describe the methodology and results of these studies.

Regarding structural analysis of the narratives, Canali et al. (2021) have investigated global coherence in a group of people with GD and in a group with SUDs. The qualitative analysis was conducted on autobiographical narratives obtained through a semi-structured interview that explored the main phases of the addiction experience (onset, maintenance, relapse, desire, loss of control, control strategies and treatment). Specifically, narrative global coherence was assessed using an adaptation of the Narrative Coherence Coding Scheme (NCCS) (Reese et al., 2011; King et al., 2014; Race et al., 2015), which is based on the assessment of three main coherence dimensions: spatial and temporal context, chronological organization, and thematic coherence. The main findings of Canali et al. (2021) showed a low level of global coherence in both groups, especially in the narrative of desire, relapse, loss of control, and control strategies, compared to the other thematic areas. Moreover, Canali et al. (2021) included in the analysis of narrative structure variables the evaluation of self-projection into the future, i.e., the occurrence of sentences in which the subject imagines himself/herself in personal future scenarios, highlighting the absence of reference to the future in both GD and SUD. With regard to narrative complexity, Rowlands et al. (2021) have investigated the association between narrative complexity and recovery in a group of people who misuses drugs or people in recovering who were administered the Life as a Film (LAAF) interview (e.g., Canter and Youngs, 2015). The Narrative Complexity index was obtained by counting the presence/absence of distinct characters and related details, the presence/absence of events, e.g., relevant past personal episodes, and the presence/absence of “psychological ideas,” as emotions or thoughts; a 12-item Recovery Inventory (RI) scale was used to assess recovery. The main result showed a positive association between the Narrative Complexity Index and the Recovery Score, particularly in the category of psychological ideas (Rowlands et al., 2021).

With regard to agency, the study by Canali et al. (2021), in addition to assessing global coherence, also conducted a linguistic analysis of some personality-related variables, including agency/passivity. Specifically, agency was assessed by counting the occurrence in the narratives of action verbs, self-efficacy markers such as “I did,” and choice indications for each thematic area in both groups (GD and SUD); passivity was assessed by counting sentences with reference to automatic behavior or passive verbs. They found a higher percentage of agency in the narration of the maintenance (chronicity) phase of the disorder compared to the other thematic areas, and greater passivity in the narration of the definition of addiction and loss of control in both groups (Canali et al., 2021). Agency was also assessed in a study by Altavilla et al. (2020), based on a linguistic investigation that highlighted specific aspects underlying self-determination and self-efficacy processes. Through a textual analysis carried out with the software LIWC (Pennebaker’s Linguistic Inquire and Word Count), personal narratives about different stages of the addiction experience (onset, maintenance, relapse, desire, loss of control, control strategies and treatment) were examined in a group of 30 people with GD. The use of pronoun-related words, i.e., the first-person singular pronoun or other persons, was counted as an indication of agency. The results showed that narratives about the definition of addiction and the stages of relapse, craving, and loss of control contained fewer first-person singular words than narratives about onset, maintenance, control strategies, and treatment. Finally, the study by Rowlands et al. (2019) investigated agency theme in a group of 32 participants who previously misused substances or in recovery, using the LAAF interview to elicit personal narratives, and assessed the outcome of the rehabilitation using the RI. Specifically, the narrative material was coded by independent evaluators for the presence/absence of agency theme by assigning a total score from 0 to 3, obtained through the detection of three agency-related themes: effectiveness, empowerment, and self-mastery. A correlation analysis to assess the relationship between agency and recovery was performed revealing a positive association between the presence of agency in personal narratives and RI score (*ibid.*). These findings were confirmed by a second exploratory study of the authors Rowlands et al, (2020), which showed a transition to low levels of agency in narratives related to substance-using identity to higher levels of agency in narratives related to recovery identity.

The study by Altavilla et al. (2020) also included a quantitative analysis of emotion-related words, i.e., positive and negative emotions, in autobiographical narratives, and showed that people with GD used a higher percentage of negative emotion-related words than positive ones, particularly when narrating the definition of addiction, maintenance, and loss of control phases, compared to other topic areas. They also counted the use of tenses (past, present, and future) in all thematic areas of the narratives, showing a lower percentage of future tense words than present and past tense words.

It is important to note that, within the framework of narrative identity, other research in recent years has explored how people with an addictive disorder narrate about their life story. However, this research has mainly focused on the mere identification of the main thematic areas through a bottom-up analysis, for example, by starting with narrative material to explore emerging themes, both of narratives about the experience of addiction, e.g., awareness of the disorder in people with GD (Rogier et al., 2020), and about recovery from GD and SUDs: e.g., on identity change through a redemption narrative framework (Stoneberg, 2022); on narratives exploring social identity transition (Webb et al., 2022); on the transition process from recognizing gambling as a problem to recovery and the role of experiences, perceptions, and social factors (Vasiliadis and Thomas, 2018); on the change of narratives of people in recovery from GD (Reith and Dobbie, 2012) and on the behavioral trajectories of people with GD’s careers (Reith and Dobbie, 2013); finally, less recently, on autobiographical narratives of people in recovery from various addictive disorders (Hanninen and Koski-Jannes, 1999). Regarding narratives of the disorder, for example, Rogier et al. (2020) quantitatively and qualitatively analyzed personal narratives of people with GD by providing a matrix of simple units present or absent in the narratives and by organizing these units into specific clusters, showing that five thematic clusters were particularly relevant in the narratives of people with GD: “gambling as dissociation, materialistic thinking, escape from social difficulties, awareness, and closeness.” Regarding recovery narratives, a study by Webb et al. (2022) examined identity changes in personal narratives collected through audio/video interviews and diary narratives of six participants in recovery from SUD at different stages of their recovery process. The narrative material was analyzed using a framework analysis, which consisted of identifying major themes (e.g., “belonging” or “confidence”) based on overarching themes of social identity statements (“staying safe/passive, exploring/transitional, and self-determinating/agentic”) and grouping them into categories (e.g., “needed” or “the real self”). Results showed a general increase in agency-related themes across stages of recovery in 5 out of 6 patients (*ibid.*).

Finally, other studies focused on the discourse strategies [e.g., the use of subjects self/other and objects in personal narratives of SUD (Sibley et al., 2020); or the use of metaphors (e.g., Shinebourne and Smith, 2010; Malvini Redden et al., 2013) used by subjects to narrate their personal experiences, particularly in relation to their social identity].

Out of a total of 18 studies mentioned in this section, we focused primarily on 9 studies that provided crucial insights into the most relevant aspects of narrative identity. An overview of the methods and findings of these main studies is provided in Table 1: 4 studies on the specific narrative variables (Rowlands et al., 2019, 2021; Altavilla et al., 2020; Canali et al., 2021); 5 studies on thematic analysis (Hanninen and Koski-Jannes, 1999; Reith and Dobbie, 2012; Rogier et al., 2020; Sibley et al., 2020; Webb et al., 2022). These investigations help to highlight the relevance of studying autobiographical narratives, particularly the mechanisms underlying how people organize their life stories, as a tool for exploring multiple aspects of the self in addiction.

**Table 1:** Overview of the investigations on narrative Identity in addictive disorders.

Reference	Participants: N, gender, mean age, other information	Diagnosis of addiction	Assessment	Main analyzed variables	Main findings relevant for narrative identity
Webb et al. (2022)	6 (4 males, 2 females) in recovery	SUDs (alcohol and/or drugs)	Identity narratives: audio and video interviews, diary-stiles narratives from early to later stages of recovery	Identity transition related to super-ordinate themes: passive, transition, agentic	Growth of agency-related themes in the later stages of recovery: from early-stage dependence, through exploration and risk-taking, to self-determining in lasts stages.
Canali et al. (2021)	30 with GD (24 male and 6 females, mean age 46.63 ± 9.08); 18 with SUDs (6 male and 2 females, 41.39 ± 6.83)	GD and SUDs	Personal narratives: Semi-structured interview on 8 main thematic areas about addiction experience (onset, maintenance, relapse, desire, loss of control, control strategies and treatment)	Global coherence Agency / passivity Future	Global coherence in GD and SUDs in desire, relapse, loss of control, and control strategies < other thematic areas. Agency in GD and SUDs in chronicization phase > other thematic areas. Passivity in GD and SUDs in the definition and loss of control > other thematic areas. Absence of utterances with future projection in both groups.
Rowlands et al. (2021)	32 (9 females, 23 males, mean age 40) who previously used substances and in recovery	SUDs	Personal narratives: Life as film (LAAF) interview. 12-item Recovery Inventory (RI)	Narrative complexity (NC): characters, events, psychological ideas Recovery index (RI)	Positive correlation between NC and RI, where a greater NC index was associated with a higher RI score especially in the category of psychological ideas.
Altavilla et al. (2020)	30 (24 males and 6 females; mean age: 46.63 ± 9.08)	GD	Personal narratives: Semi-structured interview on 8 main thematic areas about addiction experience (onset, maintenance, relapse, desire, loss of control, control strategies and treatment)	Positive/negative emotions-related words First person singular pronoun / other persons Tenses (past, present, future)	Negative emotion-related words > positive emotion-related words. Negative emotion-related words in the definition, the maintenance, and loss of control > other thematic areas. First-person singular pronouns in the definition, relapse, desire, and loss of control < the onset, maintenance, strategies of control, and treatment. Future tense-related words < past and present tense-related words
Rogier et al. (2020)	15 males (mean age 50.01)	GD	Personal Narratives: Semi-structured Interview from Indiana Illness Psychiatric Interview (IIPI, Lysaker and Lysaker, 2002)	Awareness of the disorder	Five thematic domains: Gambling as dissociation, Materialistic Thinking, Escape from Social Difficulties, Awareness, Closeness.
Sibley et al. (2020)	27 (13 females, 14 males, mean age from 25 to 59)	PWUDs (People Who Uses Drugs)	Personal Narratives: Semi-structured in-depth interview	Discourse strategies: subjects (e.g., self, and other), and objects Thematic coding: interpretative repertoires, grammatical and stylistic resources (e.g., metaphors)	Three subjects’ positions within discourses: (1) The addict as a Victim, (2) the addict as a good Samaritan, and (3) the addict as motivated for change.
Rowlands et al. (2019)	32 (23 males, 9 females, mean age 39.85) in recovery, and who previously used substance	SUDs	Personal narratives: Life as film (LAAF) interview. 12-item Recovery inventory (RI)	Agency and communion. Rehabilitation	Positive association between the presence of agency and communion themes and scores on the RI. Thematic analysis: (1) low agency, low communion with low recovery scores; (2) low agency, high communion with moderate recovery scores; (3) high agency, low communion with moderate recovery scores; (4) high agency, high communion with high recovery scores.
Reith and Dobbie (2012)	31 (8 females, 23 males, age range 20–75)	GD	Personal Narratives: Semi-structured interview for 3 times in 3 years on addiction and recovery	Thematic content: ‘Gambling Self’, ‘paths toward recovery’	Main themes: ‘disordered identities: dual selves’, ‘identities reverting’, ‘new roles in the future: moving away from gambling”.
Hanninen and Koski-Jannes (1999)	51 (22 males, 29 females) in long terms recovery, and who previously smoked	SUD, GD	Written Narratives of recovery: Interview	Story types with regard to emotional, causal, moral and ethical themes	Five story types: ‘AA story’, ‘growth story’, ‘co-dependence story’, ‘love story’ and ‘mastery story’.

SUD, substance use disorder; GD, gambling disorder.

## 4 Discussion

Addiction, both behavioral and substance-related, has traditionally been viewed as a neurobiological disorder characterized by compulsion and loss of voluntary control, with a pronounced impact on decision-making processes. Contrary to this view, in recent decades some authors have suggested that a core feature in people with an addictive disorder is related to a disruption of the patient’s structure of the self, resulting in an altered sense of self, and that this factor may underlie their maladaptive emotion regulation strategies, dissociative phenomena, and poor self-efficacy (Keane, 2002; Levy, 2006; Reith and Dobbie, 2012; Dill and Holton, 2014; Lewis, 2015). In this paper, we have shown that a privileged way to study the self is through the lens of narrative. In this view, an integrated and stable self is associated with a coherent life story that connects an individual’s internal and external experiences into a unified sense of self over time and through which people give meaning to their existence (McAdams, 1996; Singer, 2004). From this perspective, studies of various psychopathologies, such as schizophrenia and personality disorders, have analyzed the ways in which individuals construct and narrate their personal life stories in order to address important aspects of the processes underlying the structure of the self (e.g., Adler et al., 2012; Allé et al., 2015). Research has shown that certain narrative variables are particularly relevant in elucidating these processes: structural narrative variables, such as global coherence and complexity; thematic variables, such as agency; and affective dimensions, i.e., assessments of the emotional content of the narrative. Although, as we have seen, only 4 studies have applied narrative dimension analysis to the study of addictive disorders by focusing on these variables, we believe that, along with the other thematic analysis, they provide important insights into the debate over the characterization of addictions. Indeed, the findings of these studies seem to converge with the main themes emerging from the bottom-up analyses of the narratives of people with addictive disorders, that revealed a disorganization of the structure of the self and a lack of agency.

In light of the mechanisms that we have shown to underlie the development and functioning of narrative identity, some features of the autobiographical narratives of people with an addictive disorder can be linked to difficulties in maintaining an integrated sense of self. One aspect that emerged from the analysis of the autobiographical narratives of both people with GD and with SUD concerns global coherence, i.e., the property associated with the ability to organize personal episodes into a unified representation by identifying causal, motivational, and thematic links between them (Canali et al., 2021). Canali et al. (2021) suggest that the low level of coherence, particularly in the narrative of craving, may reflect a difficulty in conceptualizing the affective dimension due to dissociative phenomena. In fact, during craving, the individual seems to live a strong emotional dysregulation experience in which she/he has the urgency to implement regulatory strategies to reduce distress. Moreover, an impairment in the ability to coherently organize experiences may reveal difficulties not only in affective processes, but also in the cognitive processes related to this ability. In this sense, since narrative coherence is related to the ability to establish temporal and causal links between events in order to provide continuity to the narrative plot (e.g., Reese et al., 2011), the processing of temporality represents a central element for the narrative dimension (Suddendorf and Corballis, 1997; Corballis, 2011), which has been studied in other clinical populations, such as autism (Ferretti et al., 2018; Marini et al., 2019). Interestingly, as we mentioned, in both the Canali et al. (2021) and Altavilla et al. (2020) studies, the analysis of linguistic markers related to temporal factors, especially to self-projection into the future, i.e., the self-referential ability to imagine oneself in personal future scenarios (Atance and O'Neill, 2001; Buckner and Carroll, 2007), revealed an absence of reference to the future, showing that people with an addictive disorder failed to integrate their experience with possible self-based future scenarios different from the addiction condition.

Difficulties in processes related to the elaboration of the overall narrative structure in addictive disorders could also be indicated by a lack of narrative complexity (Rowlands et al., 2021). For example, the study by Rowlands et al. (2021) found a low percentage of narrative complexity indicators related to psychological ideas, i.e., markers of reflective and cognitive processes, suggesting a reduced level of cognitive complexity and possible difficulties in reflecting flexibly on emotions and thoughts. Since reflective abilities, i.e., high-level cognitive processes involving “knowledge of the nature of experiences which give rise to certain, beliefs and emotions” (Fonagy and Target, 1997, p. 680), appear to play a crucial role in self-organization, a link with the maladaptive regulatory strategies that characterize addiction can be hypothesized. Consistent with this finding, Rogier et al. (2020) have identified the materialistic thinking domain in their cluster analysis, namely a prevalence of reference to material goods such as money rather than emotion and cognitive elements, suggesting people with GD tend to use concrete thinking more than affective and meta-representational processes.

Findings on agency in the autobiographical narratives of people with an addictive disorder may help to explain important aspects of the disorder. In the study by Canali et al. (2021), an increased use of linguistic markers associated with agency was found in both people with GD and with SUD when narrating the maintenance phase of the disorder, whereas a greater use of passivity was observed when narrating the loss of control and when defining addiction. Interestingly, although the first finding seems to contradict the prevailing biomedical view of addictive disorders, which suggests people with an addictive disorder do not feel capable of initiating and controlling their actions (e.g., Leshner, 1997; Adinoff, 2004), the second finding seems to be consistent with it, suggesting that the actions they described were perceived as automatic. The same study reported that both people with GD and with SUD tended to refer to external motivation, especially when talking about the strategies they used to control their addictive behavior (Canali et al., 2021). To explain this finding, it can be hypothesized that this apparently contradictory way in which patients report their actions (they stated that they were both active and passive subjects of their actions) may reflect a more general disorganization in the way these individuals retrieve and integrate their personal lives and addiction-related experiences. Other studies based on thematic analysis have shown that people with an addictive disorder narrate themselves as being controlled by an alien self, and by failing to recognize parts of themselves, a loss of agency seems to prevail in them (e.g., Reith and Dobbie, 2012; Rogier et al., 2020; Sibley et al., 2020). For example, people who uses substances have been shown to narrate themselves as victims of external circumstances, such as those that are familiar and contextual (Sibley et al., 2020). Similarly, findings by Altavilla et al. (2020) showed that people with GD used a lower percentage of first-person singular pronouns when narrating experiences of craving, relapse, and loss of control compared to other topic areas. These findings suggest that the loss of self-determination and self-efficacy in addictions may be related to dissociative phenomena suggesting poor integration of the self (e.g., Keane, 2002). Consistently, studies examining agency in the context of recovery have shown that personal narratives characterized by the presence of agentic identity-related themes seem to be associated with better rehabilitation outcomes (Rowlands et al., 2019), and seem to play a key role in the process of identity transformation from “passive” or “addict” identity to more agentic identity in the that underpins treatment (Rowlands et al., 2020; Webb et al., 2022).

With regard to the affective dimension of narrative identity, the only study to examine emotions in the narratives of people with GD (Altavilla et al., 2020) showed a greater percentage of words associated with negative emotions in the narratives of several phases of their experiences. In line with the assumption that an overuse of words with negative valence may be associated with maladaptive coping strategies (Pennebaker and Seagal, 1999; Pennebaker and Graybeal, 2001), this finding seems to indicate a process of rumination, in which the individual passively compares the current situation with an unattained standard, which often occurs after dissociative phenomena (Bromberg, 2012; Altavilla et al., 2020).

Overall, the literature reviewed in this paper provides crucial insights into the importance of narrative approaches for understanding the organization of the self in addiction. In particular, from a clinical point of view, the detection of low coherence and narrative complexity, the presence of episodic memory gaps and loss of agency in the patient’s narration of her or his life story could guide the clinician’s interventions in the therapeutic process. Indeed, in the clinical setting, co-constructing new meanings with the patient also involves helping her/him to give order to personal experiences by finding causal links between events. The process of (re)organizing experience, for example through interventions aimed at eliciting and enhancing the patient’s reflective function, could help the patient to confront these acted and unthought states of the self. This process of self-restructuring, i.e., the recognition and integration of dissociated self states into consciousness, would help individuals to find more adaptive strategies of emotional regulation and to acquire a greater sense of continuity, coherence, and agency in their experience. For this purpose, narrative research has developed clinical treatment strategies focused on promoting a more coherent sense of self through the strengthening of narrative skills (e.g., Pennebaker, 2000; Freda, 2011). Some scholars have already studied the change of the narrative dimension during treatment in other psychopathologies, such as personality disorders (Arntz et al., 2012), and the effect of narrative-based exercises for the improvement of reflexive function in the context of higher education (Freda et al., 2014a,b) and for the reduction of psychological distress during difficult therapies (Martino et al., 2013). For example, Arntz et al. (2012) used linguistic analysis to examine descriptions of inner experiences elicited by an open-ended question at three different points in the psychotherapeutic process in individuals with personality disorders and in a control group of non-patients. The main findings showed a change in the use of negative and positive emotion words, first-person singular pronouns, and verb tens during treatment, with a reduction in negative emotion words predicting a better outcome. Overall, also in addictive disorders the restoration of identity as a person in recovery, the overcoming of the feeling of having alien parts of oneself, loss of self-governance, and unintegrated self-states (Weegmann, 2010; McConnell, 2016; McConnell and Snoek, 2018) could be facilitated through similar narrative-based approaches.

### 4.1 Future directions

The topics addressed in the current review pave the way for further investigations providing fruitful insights both from a methodological and conceptual point of view. For example, the line of investigation which highlighted in the analysis of linguistic markers an absence of reference to the future in people with GD and SUDs (Canali et al., 2021) may encourage further exploration of the temporal dimension in personal narratives in addiction. A possible way could be eliciting self-projection in time using specific questions in the interviews, aimed at exploring the already observed difficulty in making intertemporal choices involving temporal discounting (e.g., McCarroll, 2019), which implies self-control and anticipation of future outcomes that favor non-impulsive choices of delayed gratification in pursuit of greater rewards (Reynolds, 2006; Andrade and Petry, 2012; Smith et al., 2014; Nigro et al., 2019).

A further aspect is worth being examined concerns the difficulty in emotion regulation in addictive disorders and the link between emotion dysregulation and dissociative phenomena as a maladaptive strategy to cope with distress (Keane, 2002; Reith and Dobbie, 2012; Gori et al., 2016; Rogier et al., 2021a,b), which have been discussed above. Given the relevance of this emotional dimension for narrative identity, the way in which people with an addictive disorder emotionally narrate their personal experiences is worthy of further investigation.

From a methodologically point of view, the analyses used in the reviewed studies to examine personal narratives of people with an addictive disorder have some relevant strengths and some important limitations that future research must take into account. As for strengths, both the quantitative, qualitative, and thematic analyses add value to the detection of psychological processes involved in the construction of autobiographical narratives that may not emerge from more systematic and restrictive analyses. Thus, they provide a more comprehensive account of the experience of addiction. Notwithstanding, most of the studies used semi-structured in-depth interviews to elicit personal narratives, which were not validated, and internal reliability (of both measures and coders) was not calculated; they also lacked control groups for comparison and did not accurately control for social and psychological factors. Moreover, most studies had small sample sizes and an unbalanced gender distribution. From a conceptual point of view, it should be also noted that the few studies that have examined the narrative dimension of addictive disorders did not adopt a uniform definition of narrative, nor did they converge on the same factors responsible for the construction of personal identity. For example, Mihailov et al. (2021) pointed to the role of relational elements in narrative identity construction: in this perspective, the autobiographical story is also mediated by the relationship with others, so that others’ stories about us contribute to narrative identity formation. Given the relevance of social dynamics in addictions (e.g., Matthews et al., 2017; Pickard, 2020), relational elements of narrative identity should be investigated in these clinical populations.

## 5 Conclusion

This paper critically reviewed 9 studies that have explored narrative identity in people with addictive disorders. By showing that narrative may represent a privileged lens for studying the self, we emphasized the centrality of certain narrative variables, i.e., global coherence and complexity, agency, and affective dimensions, in revealing important processes related to the construction of a unified sense of self. Specifically, self-narratives resulting to be less coherent and with a few references to agency, seem to reflect unintegrated self-states in which dissociative phenomena, emotional dysregulation, and a loss of self-agency are maladaptive strategies for coping with distress. The investigation of these aspects has been claimed to have important implications, particularly from a clinical point of view. Indeed, the detection of low coherence, loss of agency and high negative emotionality at specific points in the autobiographical story could be useful for clinicians in the therapeutic interventions.

## Author contributions

VD: Writing – review & editing, Writing – original draft, Methodology, Conceptualization. DA: Writing – review & editing, Methodology, Conceptualization. IA: Writing – review & editing, Methodology, Conceptualization. AC: Writing – review & editing, Conceptualization. FF: Writing – review & editing, Supervision, Funding acquisition, Conceptualization.
